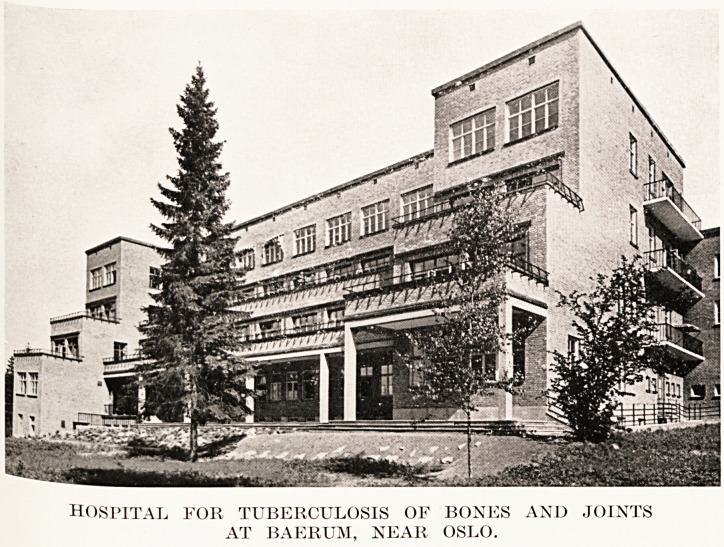# Surgical Clinics in Scandinavia

**Published:** 1936

**Authors:** A. Rendle Short

**Affiliations:** Surgeon, Bristol Royal Infirmary; Professor of Surgery, University of Bristol


### SURGICAL CLINICS IN SCANDINAVIA.

BY

A. Rendle Short, M.D., B.S., B.Sc., F.R.C.S.,

*Surgeon, Bristol Royal Infirmary;*
*Professor of Surgery, University of Bristol.*

---

During a recent visit of three weeks' duration to the Northern Capitals, I had the privilege of seeing something of the work in about ten important hospitals. It would be impossible to overrate the kindness and courtesy of one's professional brethren everywhere. They gave up hours to show me all round their wards, discussing the diagnosis and treatment of every case of interest, let me watch their operations, and at Oslo and Upsala I was invited to give a lecture to the Professor's class in Surgery.

At Helsingfors I addressed the principal Medico-Chirurgical Society in Finland, called "Duodecim," because it was founded sixty years ago by twelve students to promote the advancement of medical teaching in the Finnish tongue. For a long time there were never more than twelve, but now the membership has grown to about a thousand medical practitioners. The numbers present at the meeting, the preliminary proceedings, and the general deportment were exceedingly like that at a gathering of our Bristol Society. I spoke in English, but I doubt if many of them understood what I said. I thought a translator would have improved matters, but none was forthcoming. They had never had an English lecturer before, and I did not see the names of any English surgeons in the visiting book of any of the Helsingfors hospitals, though there were plenty in the Swedish books.

At the Elim Krankenhaus in Hamburg Dr. Hollenbach did a toxic goitre under a local anæsthetic, pantocaine, given with pressure of 1½ atmospheres by means of a screw syringe and very long rubber tube. The anæsthesia comes on immediately, is very good, and there is less bleeding than usual. Professor Holst at Oslo did a beautiful operation for a very difficult stomach case under spinal anæsthesia.

I also saw a hospital at Baerum (near Oslo), twelve miles out in very pretty country, for 100 cases of tuberculosis of bones and joints. I expected to find all children, but to my surprise there were 75 per cent. of adults, including four or five patients with sacro-iliac tuberculosis, treated by the Smith-Petersen operation. The hospital is ideal for sun-bathing in the summer, and well equipped with X-rays and ultra-violet light. I was informed that surgical tuberculosis in children is becoming uncommon in Norway, and that bovine tuberculosis is rare. A good many of the cases come to amputation.

At Upsala I was received with extreme kindness by Professor Nystrom. He does nearly everything under local or spinal anæsthesia, at which Scandinavian clinics are very skilful. High spinal anæsthesia is obtained by a fractional method, a fresh dose being given every five minutes until sensation is lost up to the costal region. The operation was on a young man with a gastric and a duodenal ulcer; partial gastrectomy with duodenectomy was performed and the anastomosis made by the Billroth I technique,

PLATE XVII

HOSPITAL FOR TUBERCULOSIS OF BONES AND JOINTS AT BAERUM, NEAR OSLO.

which is seldom used in England, but looked very good. In a case of goitre one advantage of local anæsthesia was demonstrated by making the patient say "Ja" whenever a forceps was applied anywhere near the recurrent laryngeal nerve, so that it would be practically impossible to damage it.

I was only able to see Professor Key at the Marie Hospital, Stockholm, for half an hour, but he showed me an interesting case of a condition which though rare represents a first-class surgical emergency and might occur in the practice of any doctor. A woman with chronic heart disease had been seized with sudden severe pain in the right thigh and leg and the limb became whity-blue, cold and pulseless. An embolus was extracted, about one inch long, from the femoral artery, and the limb recovered without gangrene. One has only a few hours to spare if this happy result is to be obtained. Strange to say, this was Key's forty-fifth case!

A morning at the Radium-hemmet with Dr. Berven was exceedingly instructive. He took me round about sixty cases and discussed them all. The premises are old, and next year a new Radium-hemmet is to be opened. Many of these cases are treated not by radium but by deep X-ray therapy, especially when the growth is not superficial, such as bone sarcoma, malignant glands of the neck, cancer of the prostate, breast, thyroid, etc. There was a female patient with cancer of the œsophagus who had come up to show herself, with no trace of the disease six months afterwards, but I was told that such a happy result is rare, and recurrence probable. Radium needles are still used for skin cancers, rodent ulcers, and some non-malignant conditions, also to leave in the wound after diathermy for growth of the maxilla, but not for the tongue, mouth, external genitals, and breast. Growths of the tongue and mouth, some skin cancers, and superficial growths generally, are treated by tele-radium, that is to say, several applications of a radium bomb. They have two; the one I was shown contains 5 grammes, nominal value £60,000 (less at present prices). Breast cases are all sent to the surgeon, if operable, but X-rayed before and after.

Thyrotoxicosis is treated normally by surgery, but very mild cases, and very severe ones, receive X-ray therapy.

I saw many cases with Professor Brofeldt and Dr. Nylander at Helsingfors, but the type of disease and the treatment given were much the same as in modern English clinics. Gonorrhœal joints are common; I found four amongst about one hundred patients.

The principal interest at Helsingfors was the Gynæcological Hospital, put up regardless of expense, after great trouble had been taken to think out and perfect every detail. Professor Wichmann and his lady colleague, Professor Leidenius, spent two hours showing me everything. They have bestowed much thought on the place and are justifiably proud of it. I doubt if there is such another hospital in Europe for attention to detail. Only a very few out of hundreds of small points can be mentioned here. The wards are two- or four-bedded. The walls and ceiling are painted slightly different colours to rest the eyes, and a patient recovering from an anæsthetic is always turned towards a quiet shade of green. The electric lights are so shaded that one patient can read and another sleep. The night-nurse called at night turns on a wall-light almost on the floor, so that it does not fall on the patient's eyes. When Dr. A. or B. or

C. or D. is wanted, a switch is pressed, which lights a score of lights all over the hospital; the particular light calls that particular doctor; the switch also puts a telephone through from every floor to the ward where he is required. Instead of sending a prepared plateful of food to each patient, a hot-lock wagon is sent round, and she helps herself to whatever she likes and as much as she likes. This simple improvement pleases the patient, and has cut the expenditure on food by one-third. There is a special kitchen for patients on a special diet.

Cold sterilized water is delivered all over the hospital and to each ward service-room, not by the heating system, but by forcing it through a filter. In each room there is a round iron disc in the wall, about one foot across, with a spout and tap attached, and behind the disc is an unglazed porcelain filter which is completely germ proof. It needs to be changed about once a month, but the cost is trifling. By this means there is a great saving of expenditure on fuel. The Professor remarked to me that it is really a French process used in wine making, but taken over by certain German hospitals, and he added that "when the Germans take over anything from the French, you may be sure it is pretty good."

Even the bed-pan service has been worked out on a system, to give each patient a warmed, cleansed, sterilized metal pan; they are stored on a wagon ready for use. All food and other wagons are provided with bumpers to protect the walls.

One hospital I saw was unfinished; it was as tall as an American skyscraper, I believe ten storeys. The Scandinavians have learned that it makes it much easier for the staff if they can be transported by lifts instead of having to walk immense distances many times a day on the ground level. The architecture seemed very good. The lifts were lined by the most beautiful wood I have ever seen, a blue satiny kind of birch.

One came away with the impression that British practitioners ought to pay more attention to Scandinavian methods.

## Figures and Tables

**Figure f1:**